# Clinical cases with ADHD and transgender health issues

**DOI:** 10.1192/j.eurpsy.2026.10286

**Published:** 2026-07-31

**Authors:** K. Başar

**Affiliations:** Department of Psychiatry, Hacettepe University, Ankara, Türkiye

## Abstract

**Abstract:**

The relationship between neurodivergence and gender diversity is an actively growing research interest, where many research findings suggest a stronger association than that is observed in the general population. The issue has been more extensively studied with regard to the autism spectrum features, compared to attention deficit and hyperactivity. Yet, there are some prevalence studies suggesting a higher rates of ADHD in trans and gender diverse individuals. Recent research in the mental health and clinical implication of the presence of autistic features in trans and gender diverse people indicate a major contribution of the level of executive functions in the excessive mental health burden they experience, compared to other trans and gender diverse individuals, both with respect to their experience with the minority stress and their progress in gender-affirmation. Trans and gender diverse individuals with ADHD may share a similar difficulty, though with varying severity. Furthermore, ADHD may complicate the access to the potential sources of resilience, including social support, especially if it is not recognized and managed early in life. Finally, the clinical presentation of ADHD may render the access to and execution gender-affirming health services for the individual. This may be related to the failure of the individual to follow the requirements of the assessment in the clinic; but also, sometimes more importantly, the clinician’s and social relation’s failure to confide in the authenticity of expressed identity. Mental health care professional should be familiar with the increased burden of this intersection for the individual and have the means to assist the individual in both domains concurrently.

**Disclosure of Interest:**

None Declared

